# Electronic Structure Methods for Simulating Flavin’s Spectroscopy and Photophysics: Comparison of Multi-reference, TD-DFT, and Single-Reference Wave Function Methods

**DOI:** 10.1021/acs.jpcb.4c03748

**Published:** 2024-07-29

**Authors:** Mohammad Pabel Kabir, Paulami Ghosh, Samer Gozem

**Affiliations:** Department of Chemistry, Georgia State University, Atlanta, Georgia 30302, United States

## Abstract

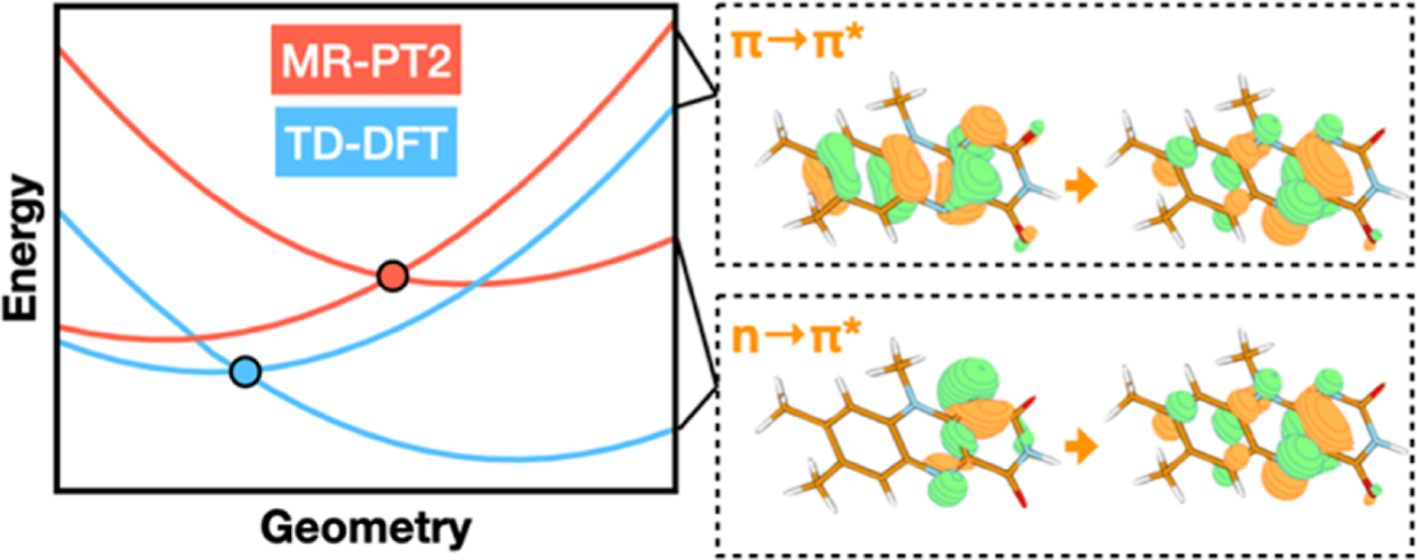

The use of flavins and flavoproteins in photocatalytic, sensing, and biotechnological applications has led to a growing interest in computationally modeling the excited-state electronic structure and photophysics of flavin. However, there is limited consensus regarding which computational methods are appropriate for modeling flavin’s photophysics. We compare the energies of low-lying excited states of flavin computed with time-dependent density functional theory (TD-DFT), equation-of-motion coupled cluster (EOM-EE-CCSD), scaled opposite-spin configuration interaction [SOS-CIS(D)], multiconfiguration pair-density functional theory (MC-PDFT), and several multireference perturbation theory (MR-PT2) methods. In the first part, we focus on excitation energies of the first singlet excited state (S_1_) of five different redox and protonation states of flavin, with the goal of finding a suitable active space for MR-PT2 calculations. In the second part, we construct two sets of one-dimensional potential energy surfaces connecting the S_0_ and S_1_ equilibrium geometries (S_0_–S_1_ path) and the S_1_ (π,π*) and S_2_ (*n*,π*) equilibrium geometries (S_1_–S_2_ path). The first path therefore follows a Franck–Condon active mode of flavin while the second path maps crossings points between low-lying singlet and triplet states in flavin. We discuss the similarities and differences in the TD-DFT, EOM-EE-CCSD, SOS-CIS(D), MC-PDFT and MR-PT2 energy profiles along these paths. We find that (TD-)DFT methods are suitable for applications such as simulating the spectra of flavins but are inconsistent with several other methods when used for some geometry optimizations and when describing the energetics of dark (*n*,π*) states. MR-PT2 methods show promise for the simulation of flavin’s low-lying excited states, but the selection of orbitals for the active space and the number of roots used for state averaging must be done carefully to avoid artifacts. Some properties, such as the intersystem crossing geometry and energy between the S_1_ (π,π*) and T_2_ (*n*,π*) states, may require additional benchmarking before they can be determined quantitatively.

## Introduction

**Figure 1:**
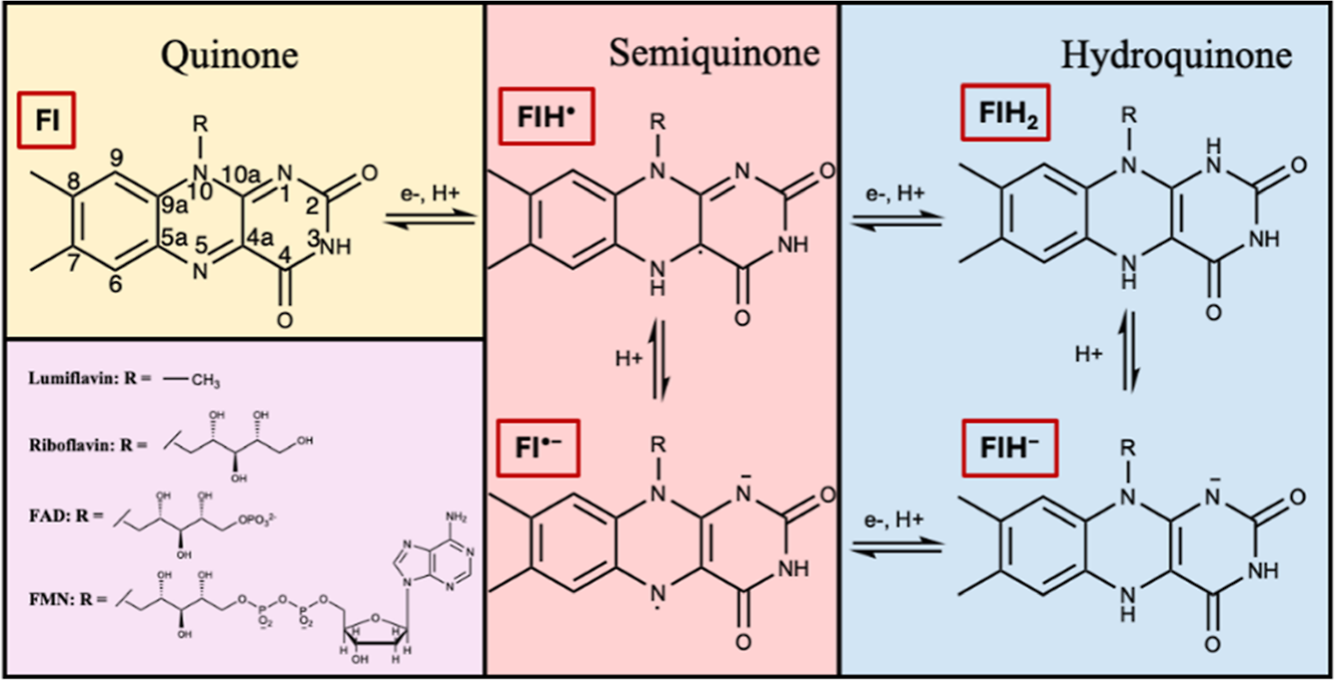
Structure of flavins in different redox and protonation states. The atom numbering is shown for the quinone (Fl) structure.

Flavins are cofactors in several classes of enzymes and in photoreceptor proteins, where they are typically bound as either flavin mononucleotide (FMN) or flavin adenine dinucleotide (FAD). Flavins are often encountered in one of three redox states: the oxidized quinone (Fl), the one-electron reduced radical semiquinone (FlH^•^), and the two-electron fully reduced hydroquinone (FlH_2_). The p*K*_a_ values of these states are 10.3, 8.3, and 6.7 in aqueous solutions, respectively.^1−3^ Therefore, the semiquinone and hydroquinone redox states are frequently also encountered in their deprotonated anionic forms, Fl^•–^ and FlH^–^, under physiological conditions (Figure 1). These redox and protonation states play an important role in either coupled or stepwise electron and/or proton transfer reactions (Figure 1). Here, we refer collectively to FMN, FAD, riboflavin, and lumiflavin (LF) as flavins, since they all share the same tricyclic 7,8-dimethyl alloxazine group.

Flavin cofactors catalyze an impressive range of chemical transformations in different enzymes and are involved in several primary and secondary metabolic pathways.^4−7^ Flavoproteins are critical to all the main energy generation metabolisms^8^ including photosynthesis,^9^ aerobic and anaerobic respiration,^10−12^ and denitrification.^13,14^ Flavins also serve as chromophores in several photoreceptors and light-responsive enzymes including cryptochromes,^15^ LOV domains (phototropins),^16^ BLUF,^17^ and DNA photolyases.^18,19^

In its oxidized form, flavin has two absorption bands in the near-UV and visible range, one band centered around 450 nm, associated with excitation to the first singlet excited state (**S**_**1**_), and a second band at around 380 nm associated with excitation to a higher singlet excited state. These absorption wavelengths are relatively insensitive to the solvent or protein environment.^20,21^ Both of those states have π,π* excitation character. Other low-lying excited states also exist in that energy range, including triplet states and singlet states, but those states have substantially smaller oscillator strengths (extinction coefficients) and do not appear in the UV/visible spectrum. Even though such dark states do not get directly excited, they can play an important role in flavins’ photophysics, as exemplified by LOV domains that undergo intersystem crossing (ISC) followed by adduct formation.^22,23^

Blue light absorption excites flavin to its S_1_ state. Higher energy (near-UV or UV) absorption also ultimately populates the same state after a series of internal conversions and vibrational relaxations, per Kasha’s rule.^24^ Therefore, flavin’s S_1_ state often acts as the launch pad for flavin’s further photophysics. From there, flavin may undergo fluorescence,^25−27^ ISC to a triplet state,^25,28^ electron transfer,^29^ or photochemical reactions, forming adducts and derivatives with neighboring amino acids.^23,30−32^ These photophysical properties have been harnessed in applications including biosensing, bioimaging, optogenetics, singlet oxygen generation, and FRET spectroscopy.^25,28,33−35^

Unlike the π,π* transitions responsible for flavins’ two UV/visible absorption bands, the energies of flavin’s low-lying dark states, especially those having *n*,π* character, can be sensitive to interactions with a solvent or protein.^21,36,37^ Since those states are relevant to flavin’s photophysics, computational modeling can be a powerful tool to study their energetics in different protein environments. However, most computational studies on flavins have focused on their ground state or on bright excited states. Several studies have shown that time-dependent density functional theory (TD-DFT) is suitable for simulating flavin’s UV/visible spectra when accounting for Franck–Condon factors.^21,37−39^ On the other hand, TD-DFT has not been as thoroughly tested for describing excited states relevant to flavin’s photophysics, including triplet, *n*,π*, or redox states.

When dealing with a manifold of excited states that are close together in energy, multiconfigurational wave function methods such as complete active-space self-consistent field (CASSCF)^40^ are suitable. In CASSCF, a full configuration interaction is carried out for select electrons and orbitals (the active space). While CASSCF misses dynamical electron correlation for electrons outside of the active space, post-CASSCF (multireference) methods can be used to correct for this missing correlation. Second-order multireference perturbation (MR-PT2) theories such as single state (SS-)CASPT2 and multistate (MS-)CASPT2 are widely used.^41,42^ More recently, to address issues with artifacts in SS-CASPT2 and MS-CASPT2 arising at near-degeneracies of the underlying CASSCF reference wave function, methods like the extended multistate (XMS) CASPT2^43^ and extended dynamically weighted (XDW) CASPT2^44^ have been developed. The former employs a treatment of the zeroth order Hamiltonian proposed as by Granovsky^45^ which removes the artifacts but alters the energy of the states of interest compared to MS-CASPT2. The latter approach, XDW-CASPT2, also addresses the artifacts but largely preserves the energies of the MS-CASPT2 states.

An alternative approach for accounting for static and dynamical electron correlation is the multiconfiguration pair-density functional theory (MC-PDFT),^46,47^ which combines favorable characteristics of CASSCF and DFT. MC-PDFT has been used in a range of applications from transition metal chemistry to mechanistic studies on biological photoreceptors.^48,49^

Several methods, including semiempirical, TD-DFT, and multireference (often, MR-PT2) methods have been used already to calculate the excited-state properties of flavins.^21,36−39,50−81^ For multireference calculations, the issue of active space selection is a recurring theme and a decision is often made that balances computational cost and computational accuracy due to the steep increase in computing time with the size of the active space. Automated protocols to select the active space have been developed by several research groups.^74,82−86^ Sayfutyarova and Hammes-Schiffer applied their automated protocol that is based on Hückel theory for the selection of a suitable active space for flavin’s π-conjugated orbitals.^74^ However, the choice of active space is also dependent on the problems and states of interest. For example, for calculating bright π,π* transitions, it is often adequate to include only π electrons and π and π* orbitals in the active space. For the calculation of dark *n*,π* states, however, including nonbonding orbitals in the active space is required. Therefore, there is still a need to tailor active spaces not only to the system but also to the problem of interest.

We present a benchmark study focused on flavin’s low-lying excited states that are relevant to flavin’s early photophysics. We focus on LF (Figure 1), a minimal flavin model that contains the spectroscopically and photochemically relevant isoalloxazine tricyclic moiety. We test several TD-DFT functionals, equation-of-motion coupled cluster (EOM-EE-CCSD),^87,88^ scaled opposite-spin configuration interaction [SOS-CIS(D)],^89^ MC-PDFT, and several MR-PT2 methods. For MR-PT2, we present the effect of modifying parameters including the active spaces and state averaging. We initially focus on excitation energies to the S_1_ excited state of each redox state. We then move on to potential energy scans (PESs) of low-lying excited states, tracing the coordinates from the Franck–Condon point (at the ground state optimized geometry) to the S_1_ (π,π*) minimum and then from the S_1_ (π,π*) minimum to the S_2_ (*n*,π*) minimum. These scans are used to probe the reliability of different methods across key geometries and state crossing points.

## Methods

### Vertical Excitation Calculations

Each of the five redox states of LF shown in Figure 1 (Fl, FlH^•^, Fl^•–^, FlH^–^, FlH_2_) were optimized in the gas phase using second-order Møller–Plesset perturbation theory (MP2) with the correlation-consistent polarized valence triple-ζ (cc-pVTZ) basis set. The optimizations were performed without the use of symmetry. In the case of Fl, FlH^•^, and Fl^•–^, the isoalloxazine ring remains planar during the optimization. However, FlH^–^ and FlH_2_ are bent at the central ring at their equilibrium geometry, exhibiting what has been referred to as a butterfly bend.^21,90−92^

Vertical excitation energies (VEEs) were computed using CASSCF, MC-PDFT, SS-CASPT2, MS-CASPT2, XMS-CASPT2, and XDW-CASPT2. Energies were also computed using TD-DFT with the B3LYP functional, which has been shown to give adiabatic excitation energies (AEEs) for the oxidized form of flavin consistent with experiments.^21,38,39,61^ The TD-DFT calculations were carried out using cc-pVTZ, while the ANO-L-VDZP basis set was used for the CASSCF, MC-PDFT, and MR-PT2 calculations.

To determine a suitable starting point for the active space, orbital entanglement calculations were carried out at the CASSCF density matrix renormalization group (CASSCF-DMRG) level of theory^86,93^ and ANO-L-VDZP basis set. Orbital entanglement figures were generated using autoCAS,^94^ and served as starting point for the selection of the active space orbitals for CASSCF and multireference calculations. The number of active space orbitals and electrons were gradually reduced based on their occupancy to test the effect of using a smaller active space on the excitation energies. The number of roots used in the state averaging was also benchmarked.

To mitigate the issue of intruder states in MR-PT2 calculations, an imaginary shift of 0.2 hartree was used.^95^ We tried using a real shift, which gave similar energies except for a few spurious spikes in the excitation energy for some geometries.^96,97^ We also tested the effect of applying a shifted zeroth-order Hamiltonian (IPEA shift) utilizing the default value of 0.25 atomic units.^98^ The IPEA shift is often used as an approximate correction for an unbalanced description of open-shell and closed shell electronic states, although it may not be needed if a sufficiently large basis set and active space is used.^99^

### Potential Energy Scans

**Figure 2:**
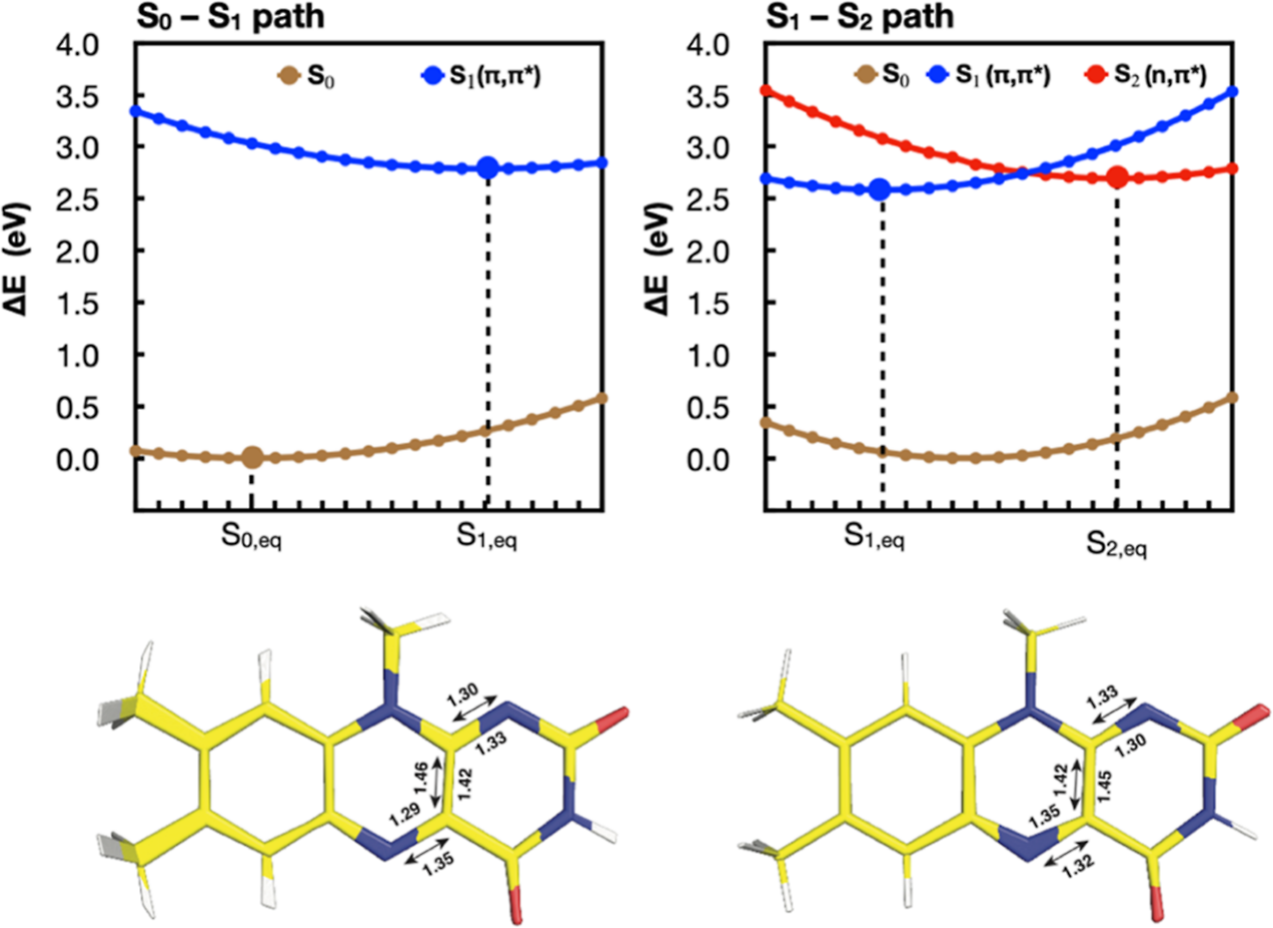
(TD)-B3LYP S_0_ (brown), S_1_ (blue), and S_2_ (red) energies along two paths used for benchmarking electronic structure methods. The S_1_ and S_2_ labels are conserved throughout the PESs, despite state crossings, to reflect their energy ordering at the ground state equilibrium geometry. The larger circles indicate the minima optimized at the (TD)-B3LYP level of theory. Smaller circles are obtained by linear interpolation and extrapolation. At the bottom of each plot, a figure is shown overlaying the structures of all interpolated and extrapolated geometries. Bond thickness in this figure therefore correlates to the extent of change in coordinates. The labels above and below the structure indicate the bond lengths at the S_0_ and S_1_ (left) and S_1_ and S_2_ (right) structures, respectively.

To generate one-dimensional PESs along coordinates relevant to flavin’s photophysics, we reoptimized the S_0_, S_1_, and S_2_ states of flavin using (TD-)B3LYP/cc-pVTZ with *C*_*s*_ symmetry enforced. We then generated two 1-D PESs by linear interpolation of Cartesian coordinates. The first PES (S_0_–S_1_ path, see Figure 2 left) connects the S_0_ optimized geometry to the S_1_ optimized geometry and therefore follows a Franck–Condon-active mode. The second PES (S_1_–S_2_ path, see Figure 2 right) connects the S_1_ optimized geometry to the S_2_ optimized geometry and therefore traces the path where the two states may potentially cross. We also added additional points on either side of each path through a linear extrapolation. In total, each path contains twenty-one geometries obtained through interpolation and extrapolation, as shown in Figure 2.

Single-point calculations along the PESs were performed using TD-DFT with the B3LYP,^100,101^ camB3LYP,^102^ and ωB97X-D^103^ functionals and the cc-pVTZ basis set. EOM-EE-CCSD and SOS-CIS(D) energies were computed with the cc-pVDZ basis set. MR-PT2 calculations along the scans were also carried out using the ANO-L-VDZP basis set. A [14,12] active space was used for the PESs, comprising two nonbonding orbitals with electron density on the N_1_ and N_5_ nitrogen atoms and five π and five π* orbitals. 3-Root state averaging was used for each symmetry and spin.

MP2 and DFT calculations were run using Gaussian 16.^104^ Multiconfigurational calculations were performed with OpenMolcas version 22.10.^105^ The density matrix renormalization group (DMRG) calculations for orbital entanglement were run using the QCMaquis interface with OpenMolcas.^106^ The resultant orbital entanglement data were analyzed using autoCAS.^94^ EOM-EE-CCSD and SOS-CIS(D) calculations were run using Q-Chem 5.4.^107^

## Results and Discussion

**Figure 3:**
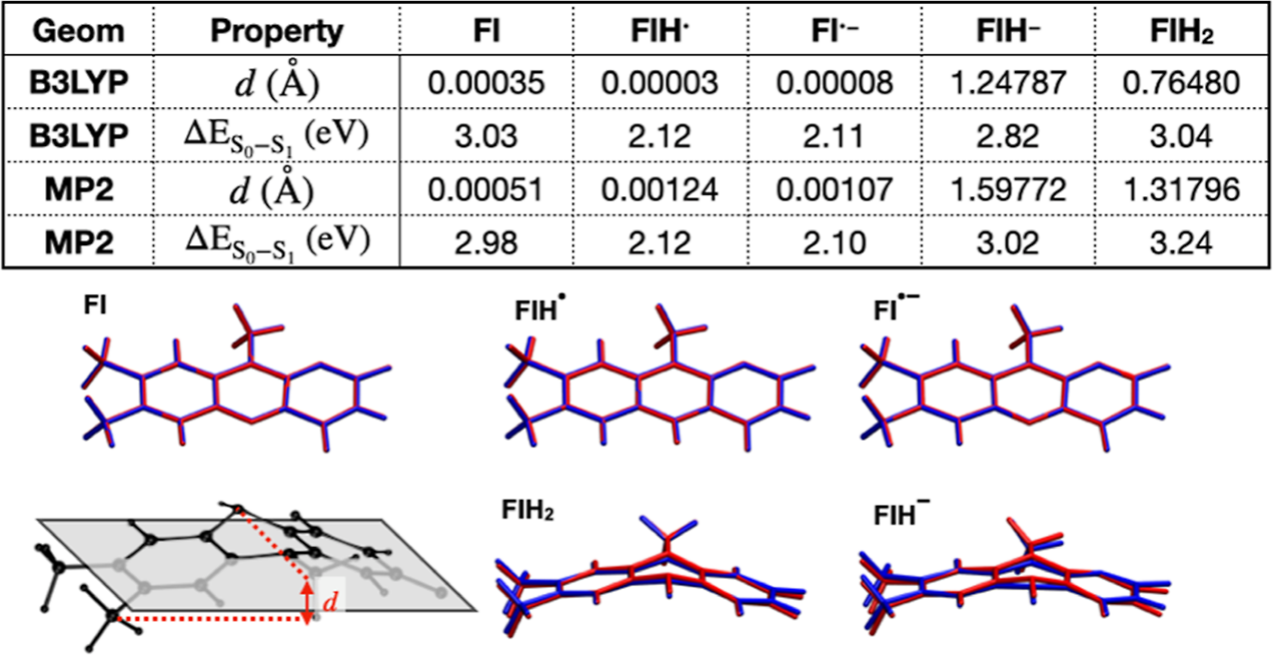
Comparison of geometries and TD-B3LYP/cc-pVTZ excitation energies of flavin in five different redox states optimized with B3LYP and MP2. The schematic at the bottom left panel indicates the definition of the parameter *d*, which is difference in the maximum and minimum positions of heavy atoms along the *z* axis when the molecules are aligned as closely as possible along the *x*–*y* plane. The other five panels show the overlay of the structure for each redox state optimized at the B3LYP (blue) and MP2 (red) levels of theory.

The geometries for Fl, FlH^•^, Fl^•–^, FlH^–^, FlH_2_ optimized at the MP2/cc-pVTZ level of theory are compared to the geometries optimized at the B3LYP/cc-pVTZ level of theory from ref (21). In Figure 3, we align the molecule along the *x*–*y* plane as closely as possible and find the maximum and minimum displacements of heavy atoms along the *z*-axis. This distance, labeled *d*, indicates the extent of the “butterfly” bending of the isoalloxazine rings in the MP2 and B3LYP geometries for the different redox states. The MP2 and B3LYP geometries are also shown superimposed in the same figure. Even though all molecules were optimized without symmetry constraints, Fl, FlH^•^, and Fl^•–^ remain planar, with only minimal deviations from planarity. The MP2 and B3LYP geometries are similar, which is also reflected in the TD-B3LYP S_1_ excitation energies that differ by no more than 0.05 eV for the two geometries. However, FlH^–^ and FlH_2_ show significantly more bending with MP2 compared to B3LYP. The distance *d* is 1.60 Å for FlH^–^ at the MP2 level of theory compared to 1.25 Å with B3LYP, while in FlH_2_, *d* is 1.32 Å with MP2 compared to 0.76 Å with B3LYP. This causes a larger difference in the excitation energies computed with the MP2 and B3LYP geometries (a 0.22 eV difference). Therefore, accurate calculations of thermodynamic quantities or excitation energies for those two reduced states of flavin may necessitate the use of geometries optimized with MP2 or a better level of theory.

**Figure 4:**
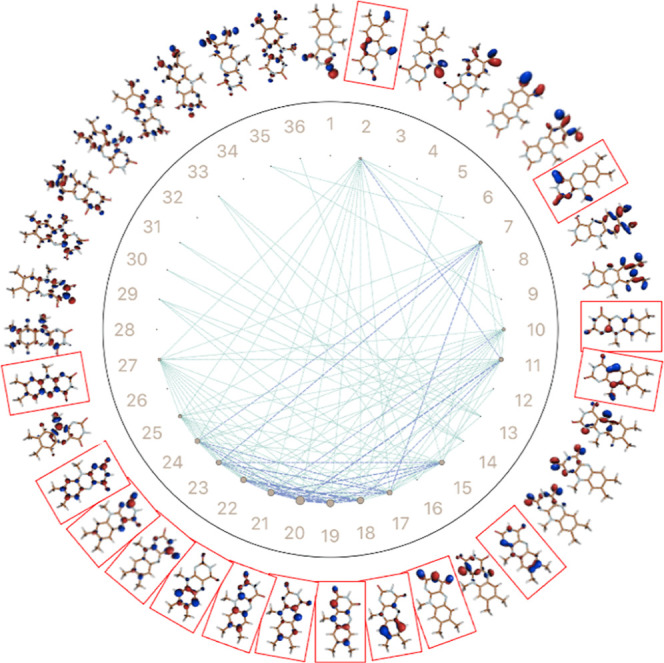
CASSCF-DMRG [38,36] orbital entanglement diagram for oxidized LF. Gray circle size represents the degree of orbital entropy while line thickness indicates mutual information between orbital pairs. Red boxes highlight orbitals with high entropy.

Figure 4 presents the orbital entanglement diagram for the oxidized form of flavin, which was generated from CASSCF-DMRG wave functions with a [38,36] active space. The diagrams feature a circular arrangement of active orbitals where strong orbital “entropy,” a term used in this context to signify a high degree of correlation with other orbitals, is indicated by larger gray circles and mutual information is indicated by the line thickness between the orbitals. A set of 15 orbitals, highlighted in the red boxes in the diagram, exhibited higher single-orbital entropies and mutual information. These orbitals comprise eight bonding π orbitals (16 electrons) and seven antibonding π* orbitals, which are shown in Figure S1a of the Supporting Information document. Consequently, we employed CASSCF with a 16 electron and 15 orbital active space as the starting point for further benchmarking.

While the DMRG-CASSCF calculations indicate the relevance of these 15 orbitals to the molecule’s multiconfigurational electronic structure, such a large active space makes routine calculations on flavins intractable. This is especially true if one is interested in looking at other excited states such as *n*,π*, which would necessitate the addition of n orbitals on top of π and π*. Therefore, we examined the effect of reducing the active space on the lowest excitation energy of flavin.

**Figure 5:**
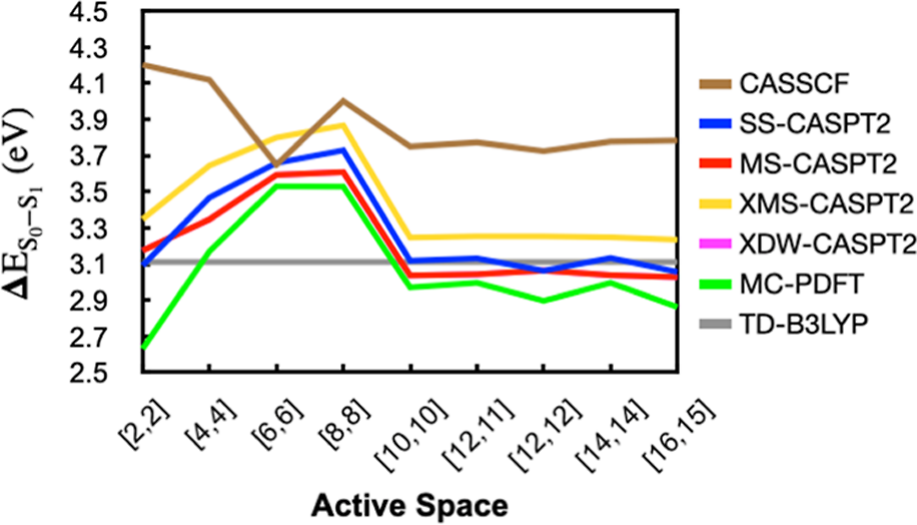
3-root MR-PT2 and 3-root state averaged CASSCF S_1_ excitation energies as a function of active space [number of electrons, number of orbitals] for LF in the oxidized form. A 0.25 IPEA shift is used for MR-PT2 methods. The TD-B3LYP excitation energy is shown as a gray line for reference. XDW-CASPT2 energies are identical to MS-CASPT2 energies in this figure.

In Figure 5, MR-PT2 and MC-PDFT excitation energies are plotted as a function of active space. 3-Root state averaging is used for the underlying CASSCF wave function in all cases. The effect of using additional roots is explored further in Supporting Information Figure S2, where it is shown that the MR-PT2 energies are relatively stable with the use of three or more states in the CASSCF state averaging. We started with a 16 electron and 15 orbital [16,15] active space shown in Figure S1a. Removing orbitals based on their occupancy had a relatively small impact on S_0_–S_1_ excitation energies down to a [10,10] active space. However, removing additional orbitals resulted in significant energy variations, indicating unbalanced electron correlation in smaller spaces. A minimal active space of [2,2] gives reasonable excitation energies for most MR-PT2 methods, as has been also shown by Udvarhelyi and Domratcheva.^53,57^ Overall, the results in Figure 5 suggest that a [10,10] active space is sufficient for accurate π,π* excitation energy calculations when using the orbitals labeled as π_1_, π_2_, π_3_, π_4_, π_5_, π_1_^*^, π_2_^*^, π_3_^*^, π_4_^*^, and π_5_^*^ in Figure S1a. SS-CASPT2, MS-CASPT2, and XDW-CASPT2 provide largely consistent energies. XMS-CASPT2 gives energies that are consistently 0.2 eV above those methods, while MC-PDFT gives energies that are around 0.1 to 0.25 eV below these methods, depending on the active space used.

**Figure 6:**
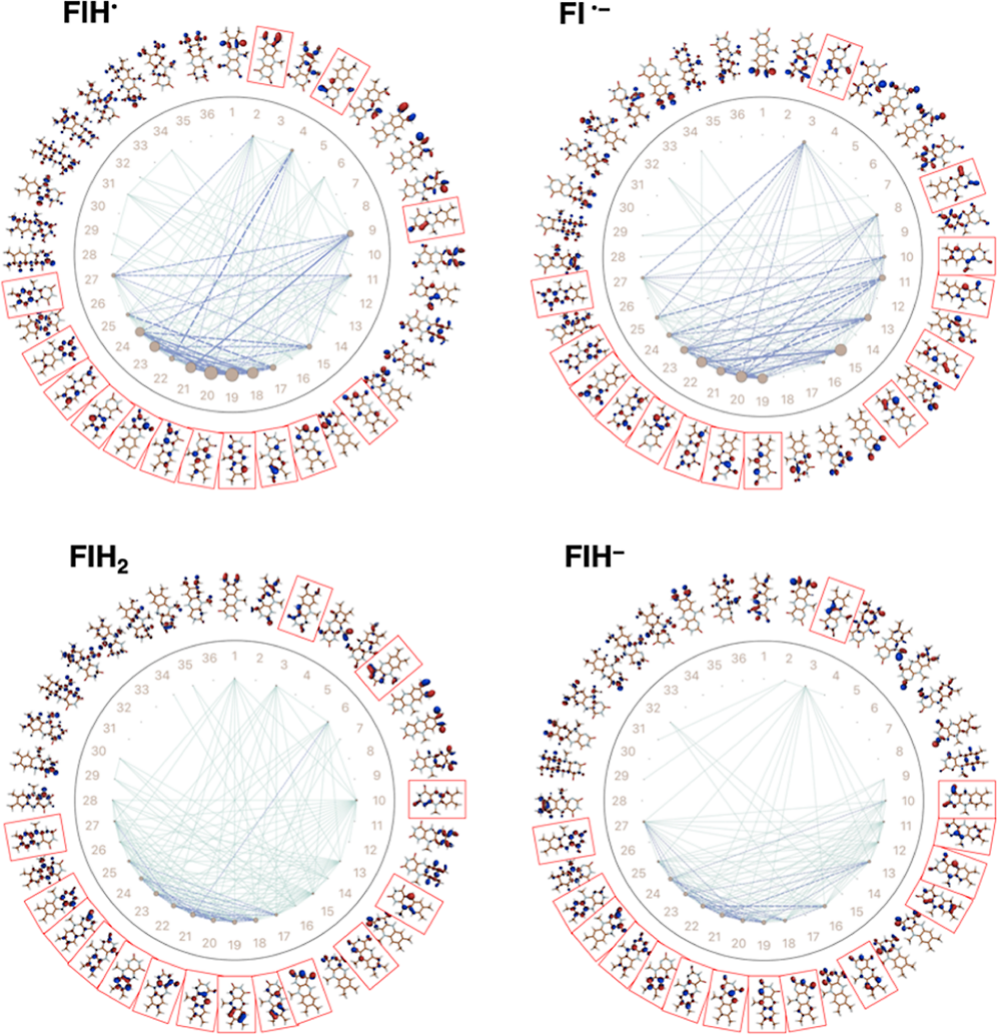
Orbital entanglement diagram for the semiquinone and hydroquinone reduced forms of LF. CASSCF-DMRG [39,36] actives spaces were used for the semiquinones and [40,36] for the hydroquinones. Gray circle size represents the degree of orbital entropy while line thickness indicates mutual information between orbital pairs. Red boxes highlight orbitals with high entropy.

In Figure 6, we show AutoCAS maps constructed from CASSCF-DMRG wave function calculations on FlH^•^, Fl^•–^, FlH_2_, and FlH^–^. The calculations for FlH^•^ and Fl^•–^ use a [39,36] active space, while those for FlH_2_ and FlH^–^ use a [40,36] active space. We find that eight bonding and six antibonding orbitals are important for both FlH^•^ and Fl^•–^. For FlH_2_ and FlH^–^, nine bonding and six antibonding orbitals with high entropy were found instead. These orbitals are highlighted in red boxes and are shown in Supporting Information Figure S1b–e along with their occupancies.

**Figure 7:**
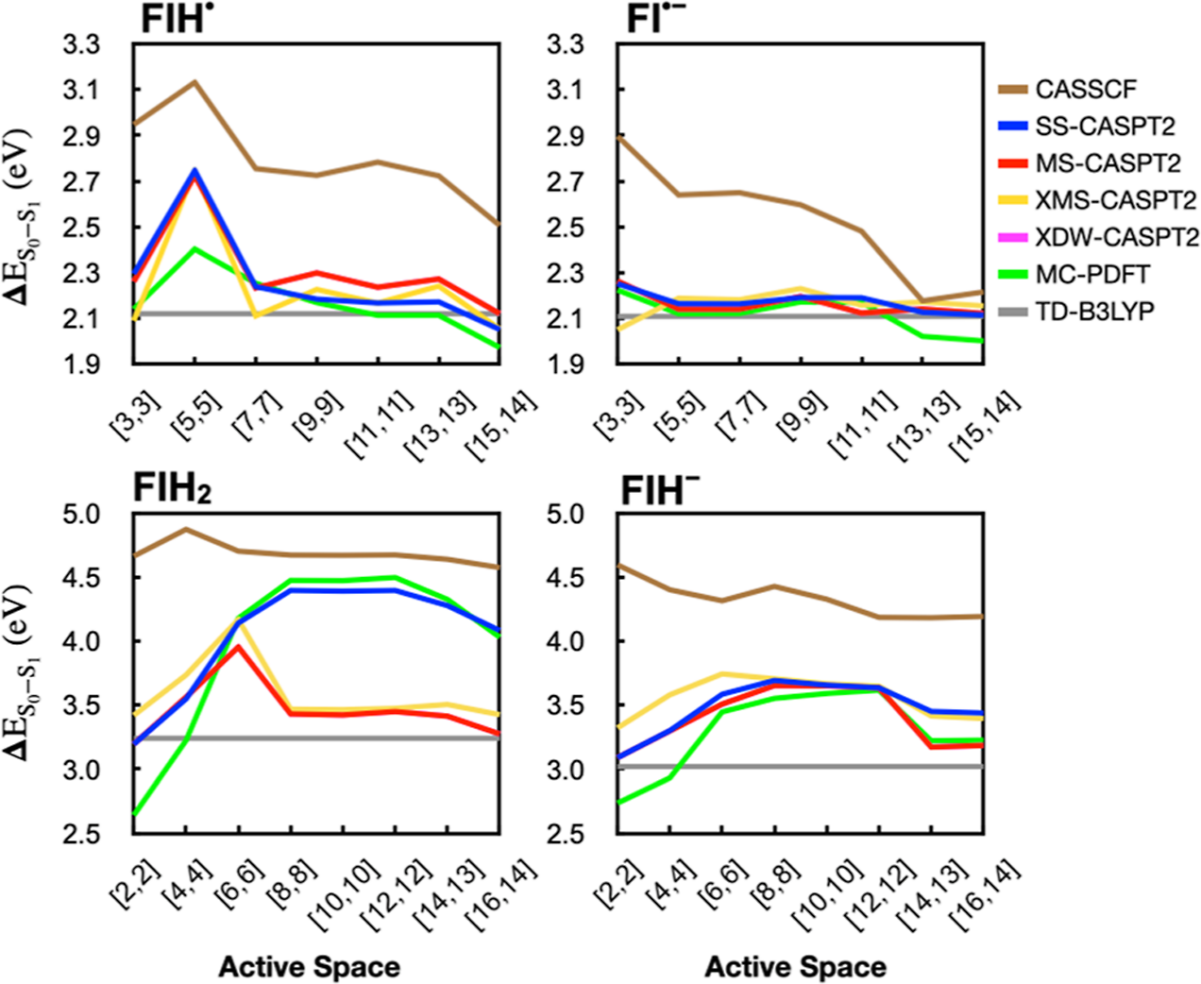
3-Root MR-PT2 and 3-root state averaged CASSCF S_1_ excitation energies as a function of active space [number of electrons, number of orbitals] for LF in different redox states. The IPEA = 0.25 shift is used for all MR-PT2 methods. The redox state is indicated above each panel. The TD-B3LYP excitation energy is shown as a gray line for reference. XDW-CASPT2 energies are identical to MS-CASPT2 energies in these figures.

We computed the excitation energies for these four redox and protonation states as a function of decreasing active space size by sequentially removing the highest occupancy orbitals, as we did for Fl. The excitation energies obtained from these calculations, along with TD-B3LYP energies, are shown in Figure 7.

For FlH^•^ and Fl^•–^, the MR-PT2 and MC-PDFT calculations with large active spaces gave results largely similar to TD-B3LYP. For FlH^•^, reducing the active space size had a limited effect down to a [7,7] active space. A minimal active space, [3,3], also gives similar results, but a [5,5] active space appears imbalanced. In FlH^–^, on the other hand, any active space [3,3] or larger gave similar energies.

In FlH_2_, and FlH^–^, which have nonplanar geometries, the convergence of the excitation energies with increasing active space size is much less smooth compared to the other states. Up to the largest active space tested, [16,14], there continue to be changes in the excitation energies. This may be attributed to the bent structure of these two redox states, which allow mixing between nonbonded or σ orbitals with π orbitals that likely results in stronger correlation between a larger set of orbitals. This can be seen to some extent in the autoCAS maps in Figure 6, where FlH_2_ and FlH^–^ have more orbitals involved in the mutual information exchange, even though the degree of entanglement is smaller for each orbital.

In FlH_2_, there is a large difference of ca. 1.0 eV between the MC-PDFT and SS-CASPT2 results compared to those calculated with MS/XMS/XDW-CASPT2. Such differences between SS-CASPT2 and MS-CASPT2 have been previously discussed^45^ and are usually resolved through the XMS or XDW extension to these theories, as appears to be the case here.

### PESs for Flavin’s Low-Lying Excited States

For the application of electronic structure methods toward studying the photophysics of flavin, it is important to move beyond single point energy calculations and to explore the energies of different methods along relevant coordinates. Here, we focus on PESs near flavin’s Franck–Condon region along two modes (Figure 2). One mode (S_0_–S_1_) connects the geometries of the S_0_ and S_1_ minima optimized at the (TD)-B3LYP/cc-pVTZ level of theory. The second mode (S_1_–S_2_) connects the S_1_ and S_2_ minima optimized at the TD-B3LYP/cc-pVTZ level of theory. The aim is to check the sensitivity of the relative energies, curvatures, and crossing points of the low-lying singlet and triplet excited states along these paths to the electronic structure method.

**Figure 8:**
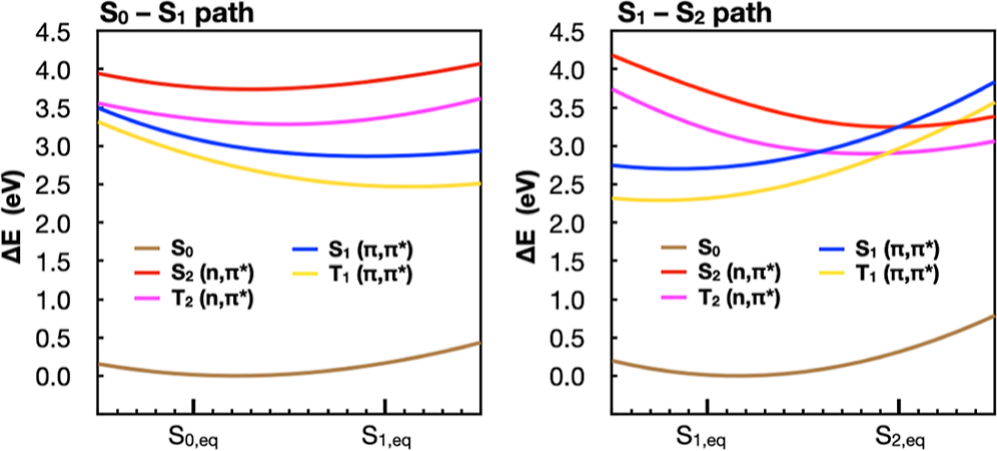
3-Root MS-CAPT2(IPEA = 0.25)[14,12]/ANO-L-VDZP S_0_ (brown), S_1_ (blue), S_2_ (red), T_1_ (yellow) and T_2_ (magenta) energies along the S_0_–S_1_ path (left) and the S_1_–S_2_ path (right).

In Figure 8, we show MS-CASPT2 energies for five states (S_0_, S_1_, S_2_, T_1_, and T_2_) along the two paths. The [14,12] active space comprises the 5 π and 5 π* orbitals consistent with the [10,10] active space from Figure 5 plus two nonbonding orbitals localized on the N_1_ and N_5_ nitrogen atoms. S_1_ is the optically active π,π* state, while S_2_ is the optically inactive *n*,π* state involving the nonbonding orbitals. The triplet states, T_1_ and T_2_, have the same electronic transitions as the S_1_ and S_2_ states, respectively. The S_1_ state crosses with both the S_2_ and the T_2_ states along the S_1_–S_2_ path.

We first focus on the S_0_ and S_1_ PESs along the S_0_–S_1_ path, since those two states are responsible for the spectroscopic properties of flavin’s first excitation band. We then shift our focus to the S_1_, S_2_, and T_2_ PESs along the S_1_–S_2_ path, since their crossings represent possible internal conversion or ISC points, respectively.

**Figure 9:**
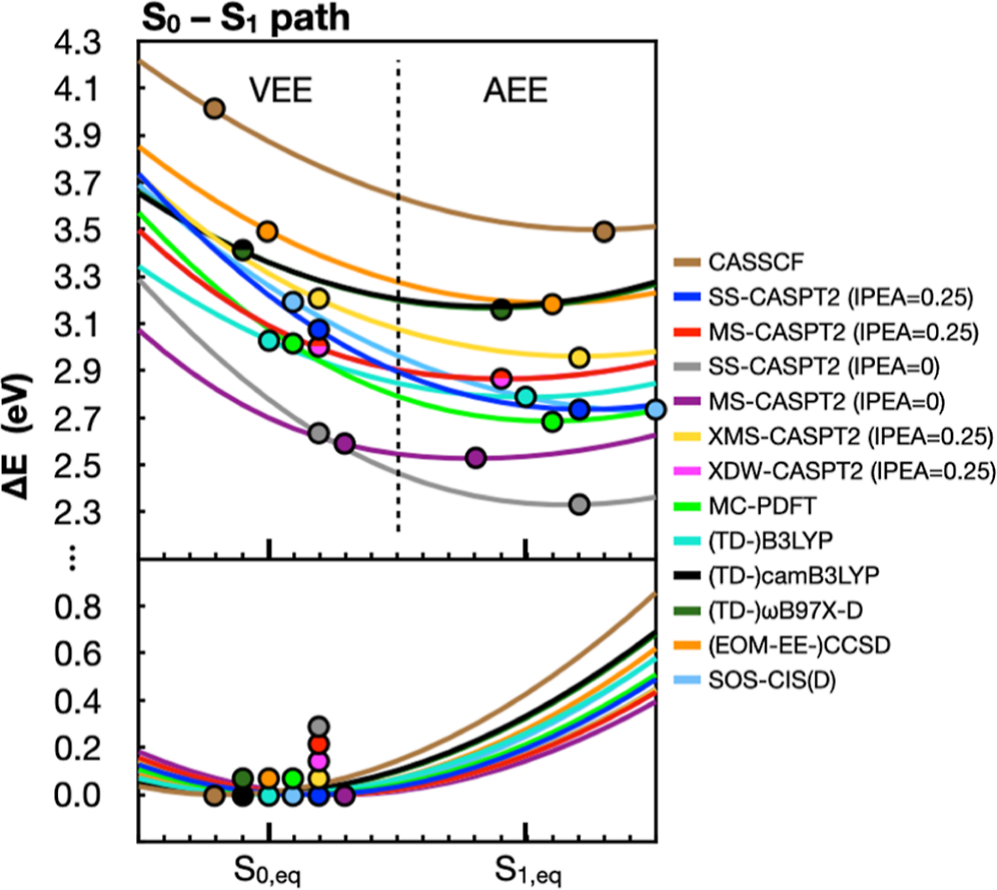
S_0_ (bottom) and S_1_ (top) energies along the S_0_–S_1_ path reported relative to the corresponding S_0_ minimum energy point along the path. Energies are shown for 3-root state averaged CASSCF, MR-PT2, MC-PDFT, TD-DFT, EOM-EE-CCSD, and SOS-CIS(D) methods, using colors indicated in the legend on the right. CASSCF and MR-PT2 energies employ a [14,12] active space. In the bottom panel, the circles indicate the interpolated geometry that has the lowest energy for each method. In the top panel, circles indicate the corresponding VEE (left side) and the AEE (right side) along this path. The latter is found as the point with the lowest S_1_ energy along the path. XDW-CASPT2 energies are identical to MS-CASPT2 energies in this figure.

Figure 9 plots the S_0_ and S_1_ energies along the S_0_–S_1_ path with different methods. For S_0_, CASSCF stands out from other curves at large distortion. This is likely due to missing dynamical electron correlation, particularly σ orbitals that become involved with bond stretching. However, even among other methods that do account for dynamical electron correlation, we find that there are differences in the positions of the S_0_ minima along the PES. CCSD is the only other method that has the same minimum point as the B3LYP-optimized S_0_ state. Conversely, camB3LYP and ωB97X-D give geometries that are further away from the S_1_ minimum and fall along the extrapolated portion of the PES. MR-PT2, MC-PDFT, and SOS-CIS(D) methods, on the other hand give minima that are closer to the S_1_ equilibrium structure. Nonparallelity errors (NPEs), which are reported relative to the XDW-CASPT2 (IPEA = 0.25) energy profile, reflect those differences as well (Table 1). NPEs are calculated as the largest deviation from the reference XDW-CASPT2 curve minus the smallest deviation from the same curve. Methods that have similar minimum points to XDW-CASPT2 and have similar curvature have small S_0_ NPEs, while methods with different minimum energy geometries display larger S_0_ NPEs.

**Table 1:** NPEs for Different Methods Calculated Relative to the Reference XDW-CASPT2 PES

	S_0_ NPE (eV)	S_1_ NPE (eV)
CASSCF	0.54	0.21
SS-CASPT2 (IPEA = 0.25)	0.08	0.42
MS-CASPT2 (IPEA = 0.25)	0.00	0.00
SS-CASPT2 (IPEA = 0)	0.03	0.37
MS-CASPT2 (IPEA = 0)	0.07	0.11
XMS-CASPT2	0.02	0.19
MC-PDFT	0.12	0.28
(TD-)B3LYP	0.23	0.10
(TD-)camB3LYP	0.37	0.18
(TD-)ωB97X-D	0.35	0.17
EOM-EE-CCSD	0.26	0.11
SOS-CIS(D)	0.20	0.41

The differences between methods become substantially more pronounced when looking at the S_1_ state, both in terms of the excitation energy as well as the shape (curvature) of the excited state potential energy surface along this path. VEEs, which are indicated with circles in the top left section of Figure 9, reflect the S_0_–S_1_ energy differences at the same position where a method’s S_0_ minimum is found. Even if we exclude CASSCF from the analysis due to its missing dynamical electron correlation, we find that VEEs vary from 2.59 eV (MS-CASPT2 with IPEA = 0) to 3.49 eV (EOM-EE-CCSD), a range of 0.90 eV. Changes in the curvature of the excited state potential energy surface are reflected by the S_1_ NPEs shown in Table 1. While some excited state methods have PESs that resemble the XDW-CASPT2 PES (S_1_ NPE within 0.11 eV), several methods stand out has having a different curvature along this path. Specifically, SS-CASPT2 (NPE = 0.37–0.42 eV), SOS-CIS(D) (NPE = 0.41 eV), MC-PDFT (NPE = 0.28 eV), and XMS-CASPT2 (NPE = 0.19 eV) all have a larger curvature compared to XDW-CASPT2. On the other hand. TD-DFT methods, which give NPEs ranging from 0.10 to 0.18, have smaller curvature.

In the top right part of Figure 9, circles are used to indicate the S_1_ minima for the different methods calculated along the S_0_–S_1_ path. The Δ*E* value at those points reflects the AEEs for the different methods. We note that those are approximate AEEs because they are not true minima but instead are the lowest energy points computed along a TD-B3LYP PES. Nonetheless, there is a marked variation in these energies, which is a result of both the differences in the VEEs as well as the differences in curvatures of the different methods. AEEs along this path range from 2.33 eV (CASPT2 with IPEA = 0) to 3.19 eV for EOM-EE-CCSD, a 0.86 eV range.

**Figure 10:**
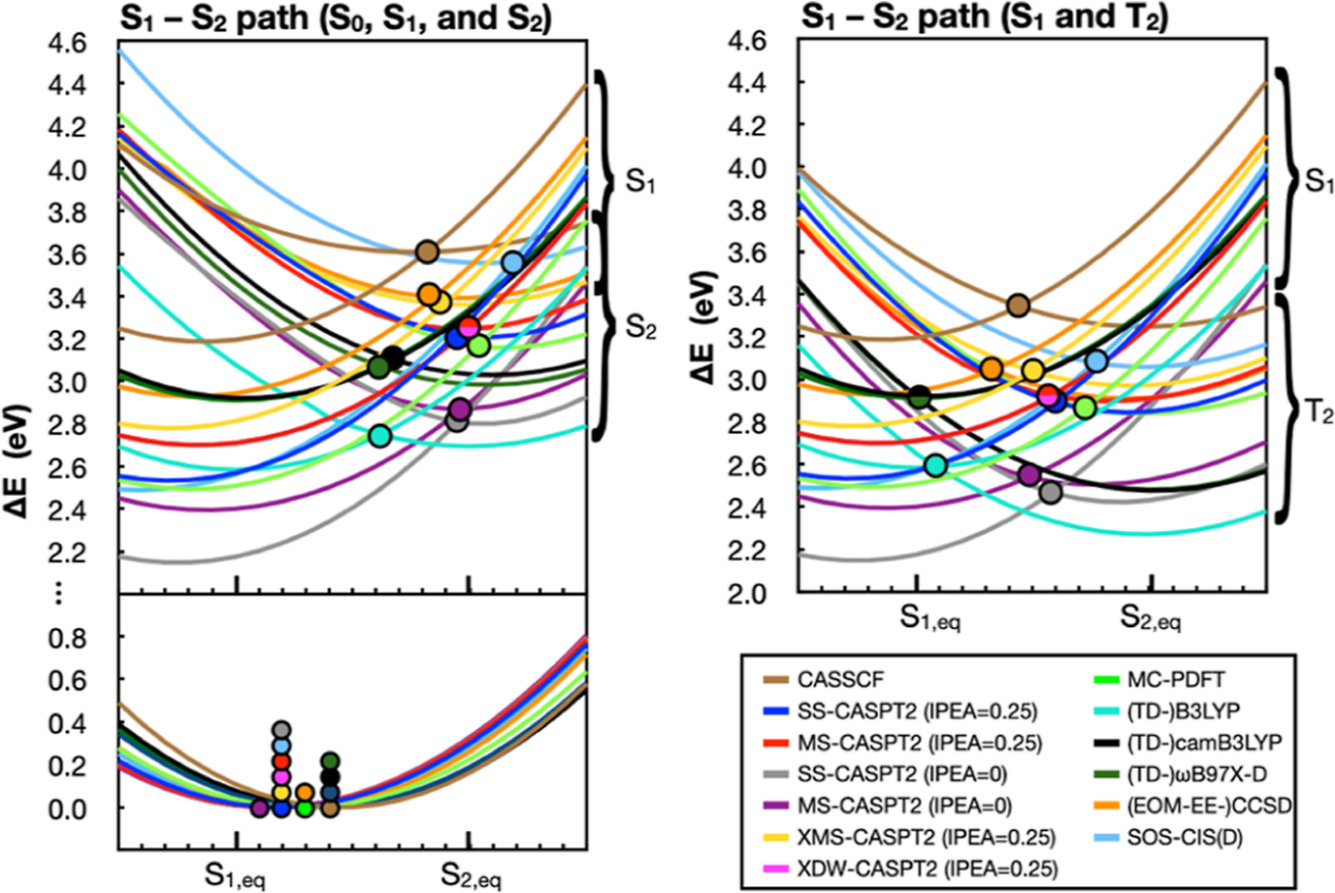
PESs along the S_1_–S_2_ path. In the left panel, we show the S_0_ (bottom left panel) as well as S_1_ and S_2_ (top left panel) potential energies reported relative to the corresponding S_0_ minimum energy point along this path. In the panel on the right, we show the S_1_ and T_2_ energies along the same path. Energies are shown for 3-root state averaged CASSCF, MR-PT2, MC-PDFT, TD-DFT, EOM-EE-CCSD, and SOS-CIS(D) methods, using colors indicated in the legend on the bottom right. MR-PT2 and CASSCF energies employ a [14,12] active space. In the bottom left panel, the circles indicate the interpolated geometry that has the lowest energy. In the tops panels, circles indicate crossing points between the S_1_ state and the S_2_ (left) or T_2_ (right) states. XDW-CASPT2 energies are identical to MS-CASPT2 energies in these figures.

In Figure 10, we plot S_0_, S_1_, S_2_, and T_2_ energies along the S_1_–S_2_ path. The circles on the S_0_ PES again indicate differences in the positions of the minima for the different methods, which all lie somewhere between the S_1_ and S_2_ geometries. In the top left panel of Figure 10, we show both the S_1_ and S_2_ states. For all methods shown, the two states cross at some point along the S_1_–S_2_ path. In all cases, the S_1_ minimum remains lower in energy than the S_2_ minimum, suggesting (at least, in the gas phase) that the S_2_ (*n*,π*) state would not get populated from the S_1_ state. Since solvents typically stabilize (red-shift) π,π* excited states and destabilize (blue-shift) *n*,π* states,^108^ we expect the same conclusion will also apply to flavins in solution. The S_1_–S_2_ crossings for all methods, indicated using circles, are geometrically closer to the S_2_ minima and often lie well above the S_1_ minima. In Table 2 we tabulate Δ*E*_S_1_/S_2__, which are energy differences between the S_1_–S_2_ crossing points and the S_1_ minima, for the different methods.

**Table 2:** Energy Barrier to the S_1_–S_2_ Crossing Point (Δ*E*_S_1_/S_2__) and the S_1_/T_2_ Crossing Point (Δ*E*_S_1_/T_2__) Relative to the S_1_ Minimum Energy^a^

method	Δ*E*_S_1_/S_2__ (eV)	Δ*E*_S_1_/T_2__ (eV)
CASSCF	0.40	0.13
SS-CASPT2 (IPEA = 0.25)	0.64	0.36
MS-CASPT2 (IPEA = 0.25)	0.55	0.23
SS-CASPT2 (IPEA = 0)	0.66	0.33
MS-CASPT2 (IPEA = 0)	0.50	0.15
XMS-CASPT2 (IPEA = 0.25)	0.59	0.26
XDW-CASPT2 (IPEA = 0.25)	0.55	0.23
MC-PDFT	0.63	0.35
TD-B3LYP	0.15	0.00
TD-camB3LYP	0.19	0.00
TD-ωB97X-D	0.15	0.00
EOM-EE-CCSD	0.47	0.10
SOS-CIS(D)	1.09	0.62

a Those barriers are estimated for different electronic structure methods along the S_1_–S_2_ path.

The S_1_/T_2_ plots (Figure 10 right) tell a different story. There, the relative energies of the S_1_ and T_2_ minima are more comparable, with some methods showing a lower S_1_ state minimum energy point and other methods showing a lower T_2_ minimum. For example, TD-DFT methods show that the T_2_ minimum is lower than the S_1_ minimum, while MR-PT2 methods show that the S_1_ minimum is lower than T_2_. The S_1_/T_2_ crossings lie somewhere in the middle between the two coordinates. As discussed by Salzmann et al. in multiple studies,^36,76,77^ solvent effects or a protein environment can play an important role in changing flavin’s photophysics by modulating the relative energies of the S_1_ and T_2_ states. However, here we also find that the relative energies of those states are sensitive to the choice of computational method.

Table 2 presents Δ*E*_S_1_/T_2__, the relative energies of the S_1_/T_2_ ISC point compared to the S_1_ minimum along the S_1_–S_2_ path, alongside the corresponding Δ*E*_S_1_/S_2__ values. We note that these crossing points were obtained from the interpolation and do not necessarily represent the minimum energy crossing points. However, the variations in these numbers for the different methods indicates either that (1) the barrier to those crossings is different for the different methods, or (2) the minimum energy crossing geometry varies from one method to another. For example, along the S_1_–S_2_ path, TD-DFT methods indicate that the S_1_/T_2_ ISC point coincides with the S_1_ minimum geometry (with a barrier of ∼0 eV) while SOS-CIS(D) gives a Δ*E*_S_1_/T_2__ of 0.62 eV. EOM-EE-CCSD gives a Δ*E*_S_1_/T_2__ of 0.10 eV while MR-PT2 methods give Δ*E*_S_1_/T_2__ values that vary from 0.23 to 0.36 eV.

When looking more generally at the trends in both Δ*E*_S_1_/S_2__ and Δ*E*_S_1_/T_2__, most MR-PT2 methods and MC-PDFT appear to treat the π,π* and *n*,π* states on a similar footing, since the crossing geometries do not change considerably from one method to the other. The IPEA shift has a large effect on the S_1_, S_2_, and T_2_ energies relative to the reference S_0_ state, but it does not have much of an impact on their energies relative to each other since all three states have similarly open-shell character. This is results in Δ*E*_S_1_/S_2__ and Δ*E*_S_1_/T_2__ barriers for the IPEA = 0 calculations that are comparable in magnitude to their IPEA = 0.25 counterparts. TD-DFT methods, on the other hand, predict lower energies for both the S_2_ and T_2_ (*n*,π*) states, give crossing geometries that are closer to the S_1_ minimum, and have considerably smaller Δ*E*_S_1_/S_2__ and Δ*E*_S_1_/T_2__ energies. EOM-EE-CCSD gives crossing geometries and Δ*E*_S_1_/S_2__ and Δ*E*_S_1_/T_2__ energies that are intermediate between those of TD-DFT and MR-PT2. SOS-CIS(D), on the other hand, appears to significantly overestimate Δ*E*_S_1_/S_2__ and Δ*E*_S_1_/T_2__ compared to the other methods. We note that EOM-EE-CCSD and SOS-CIS(D) calculations were carried out with a smaller basis set (double-ζ instead of triple-ζ), which may contribute to the difference observed.

**Figure 11:**
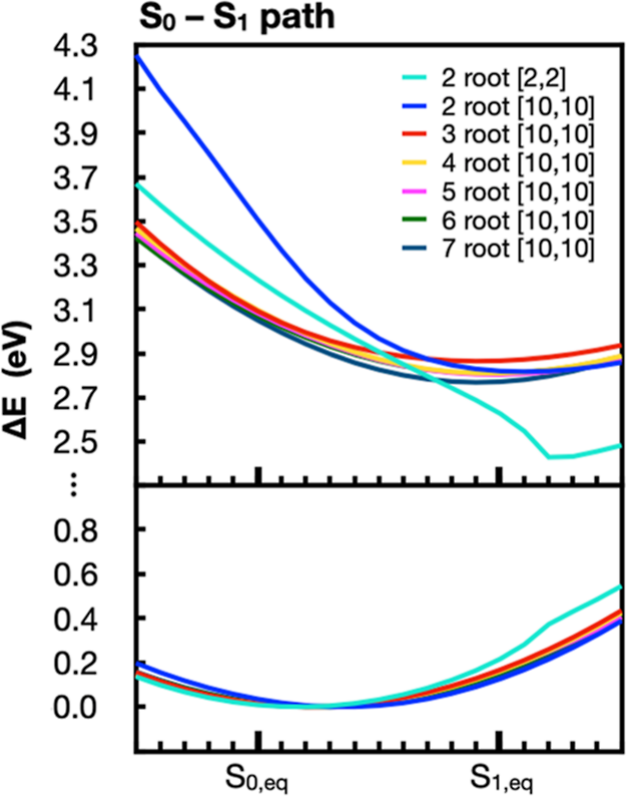
S_0_ (bottom panel) and S_1_ (top panel) energies along the S_0_–S_1_ path reported relative to the corresponding S_0_ minimum energy point along the path. Energies are shown for MS-CASPT2 with IPEA = 0.25 but with different number of roots used in the state averaging of the underlying CASSCF wave function. We also include one calculation where the minimal [2,2] active space is used instead of [10,10].

In Figure 11, we show the energy profiles computed using MS-CASPT2 (IPEA = 0.25) with different state averaging or active space along the S_0_–S_1_ path. Using 3 or more states in the zeroth-order CASSCF wave function gives comparable S_0_ and S_1_ energy profiles along this path. However, using 2-root state averaging gives a qualitatively different S_1_ energy profile. Similarly, using a minimal [2,2] active space, while successful at reproducing the VEE of flavin, has distorted S_0_ and S_1_ PESs when moving away from the S_0_ equilibrium structure. This may result in artifacts far from the Franck–Condon region.

## Conclusions

Multiple computational studies have investigated the excited-state electronic structure and photophysics of flavin. A nonexhaustive list includes refs (21, 36–39, 50–81). A more comprehensive list of computational studies can be found in a recent review article by Kar, Miller, and Mroginski.^78^ These studies employ a range of computational methods including semiempirical, TD-DFT, coupled cluster, and multiconfigurational approaches. Even among multiconfigurational studies, methodological details such as the number of roots used in state averaging or the size of the active space may differ. With the continued advancement in the use of flavins and flavoproteins for novel photocatalytic, sensing, and biotechnological applications,^31,109−115^ we expect that computational studies on these systems will continue to elucidate and guide experimental studies. In this work, we present a comparison of how different electronic structure methods describe the energetics of the low-lying excited states of flavin.

In the first part of this study, we compared S_1_ excitation energies calculated with MR-PT2 methods and those computed with TD-B3LYP for flavin in different redox and protonation states. We focus on the S_1_ state because, according to Kasha’s rule, this is the state from which a system’s photophysics and photochemistry typically occurs. An initial active space for CASSCF and MR-PT2 calculations is selected using autoCAS, but then the number of orbitals in the active space are gradually reduced to check the effect on the S_0_–S_1_ excitation energy. Here, TD-B3LYP serves as a reasonable reference since it has been well tested against experimental spectra and shown to agree well when properly accounting for Franck–Condon factors.^21,38,39,61,67^ For most redox states, MS-CASPT2 (with the IPEA 0.25 shift) agrees well with TD-B3LYP excitation energies if a sufficiently large basis set is used. For the oxidized and semiquinone forms of flavin, which are planar, the convergence of the excitation energy is reached quickly with a small or moderate active space size. In the case of the hydroquinone forms of flavin, which are bent, a larger active space is needed for MR-PT2 methods to converge. For SS-CASPT2 and MC-PDFT, convergence with increasing active space size is not achieved for FlH_2_ even when using a 16 electron and 14 orbital active space.

In the second part of this work, we compare TD-DFT, EOM-EE-CCSD, SOS-CIS(D), MC-PDFT, and MR-PT2 energy profiles along a PES connecting the S_0_ and S_1_ equilibrium geometries (i.e., tracing the Franck–Condon active mode) and along a PES connecting the S_1_ (π,π*) and S_2_ (*n*,π*) minima. Along the S_0_–S_1_ path, we find that (TD)-B3LYP is in reasonably good agreement with MS-CASPT2 (IPEA = 0.25) energies, which explains its success in simulating UV/visible spectra. However, the same is not true for the S_1_–S_2_ path, where TD-B3LYP has a significantly lower energy for the S_2_ and T_2_ states compared to MS-CASPT2 (IPEA = 0.25). TD-camB3LYP and TD-ωB97X-D, which are hybrid functionals that include a long-range correction that is missing in TD-B3LYP, appear to also have a reduced agreement with multireference methods.

While MS-CASPT2 with a minimal [2,2] active space and/or 2-root state averaging can give VEEs that are in good agreement with larger active spaces, it is not recommended for studying regions far from the Franck–Condon point.

For all methods tested, the S_2_ minimum lies above the S_1_ minimum, suggesting that it is unlikely to be involved in flavin’s photophysics. The T_2_ (*n*,π*) state, on the other hand, may be populated by ISC from the S_1_ (π,π*) state. The relative energies of these states are sensitive to the choice of computational method, and we posit that additional benchmarking is needed for a more quantitative description of ISC in flavins. Such benchmarks should ideally also test the effect of the method on the optimized geometry of the singlet and triplet (π,π*) and (*n*,π*) states.

Earlier work with Olivucci and co-workers resulted in a series of benchmark studies on a minimal three double-bond reduced model of the retinal protonated Schiff base chromophore of rhodopsins (PSB3).^116−123^ Some of this work is reviewed in ref (124). There, energies and wave functions of a wide range of methods were compared along paths relevant to PSB3’s isomerization coordinate and S_1_ excited-state dynamics. Here, we again emphasize the usefulness of moving beyond vertical energies or energy differences between two points in benchmark studies. Instead, by comparing methods along a reaction or photophysical pathway, important differences in how these methods treat different regions of a potential energy surface may emerge. For PSB3, methods that could accurately reproduce the VEE could not necessarily describe the twisted PSB3 intermediates, which either displayed strong charge transfer character or diradical character. On the other hand, a few methods could treat both the planar and fully twisted PSB3 intermediates well but deteriorated when describing intermediate structures connecting the planar and 90° twisted geometries. Ultimately, through extensive benchmarks, methods were found that have a suitable error cancellation to treat different regions of PSB3’s potential energy surface on a similar footing. This has enabled, for instance, the construction of model potentials suitable for running quantum dynamics for PSB3.^125^ Similarly, additional benchmark studies on flavin can lead to the adoption of methods that may be used routinely for studying the photophysics and photochemistry of this system in different protein environments.

## Data Availability

The main data from this work are presented in the manuscript and in the Supporting Information. Requests for additional data, including computational models and raw data files, should be directed to S.G. (sgozem@gsu.edu).

## Supporting Information Available

Cartesian (xyz) coordinate files of LF optimized in different redox and protonation states at the B3LYP/cc-pVTZ and MP2/cc-pVTZ levels of theory without symmetry constraints. Cartesian coordinates for structures along the S_0_–S_1_ path and the S_1_–S_2_ path (21 structures each) (ZIP)
Figures of the molecular orbitals included in the active space of CASSCF and MR-PT2 calculations; plots of CASSCF S_1_ excitation energies as a function of active space and number of roots used in the state averaging for LF in the oxidized form (PDF)
