# Supplementary material for: Electronic Structure Methods for Simulating Flavin’s Spectroscopy and Photophysics: Comparison of Multi-reference, TD-DFT, and Single-Reference Wave Function Methods
Source: J Phys Chem B. 2024 Jul 29;128(31):7545–57. doi: 10.1021/acs.jpcb.4c03748 (PMC11317985; doi:10.1021/acs.jpcb.4c03748)
Supplement: Supplementary file 2 — jp4c03748_si_002.pdf [file jp4c03748_si_002.pdf]

Supporting Information for:

Electronic Structure Methods for Simulating Flavin's Spectroscopy and Photophysics: Comparison of Multi-Reference, TD-DFT, and Single-Reference Wave Function Methods

Mohammad Pabel Kabir,[1] Paulami Ghosh,[1] Samer Gozem[1,*]

[1]Department of Chemistry, Georgia State University, Atlanta, GA 30302, United States.

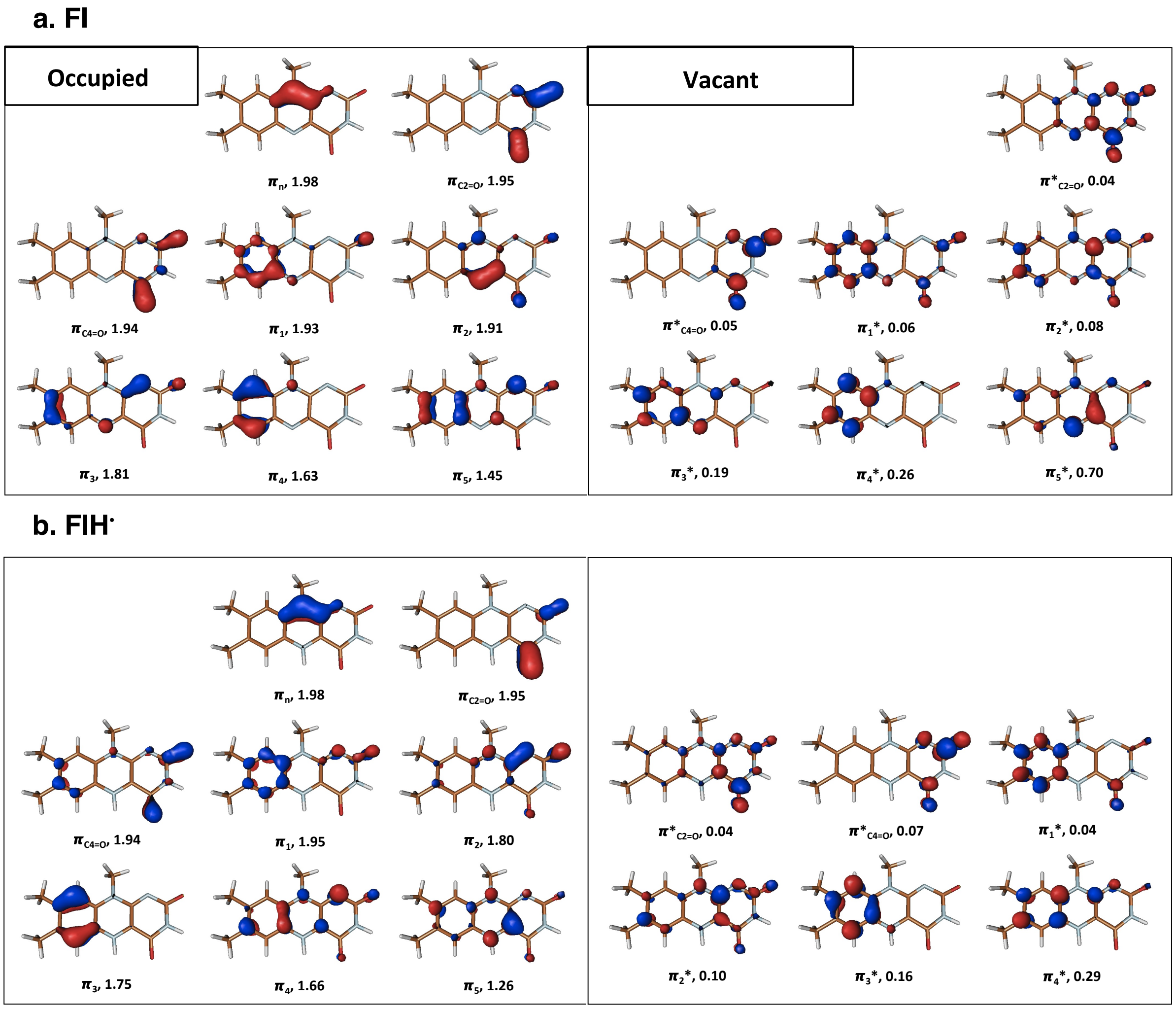
a. Fl
Occupied
Vacant
π_n, 1.98
$\pi_{C2=O}$, 1.95
$\pi^*_{C2=O}$, 0.04
$\pi_{C4=O}$, 1.94
π_1, 1.93
π_2, 1.91
$\pi^*_{C4=O}$, 0.05
π_1^*, 0.06
π_2^*, 0.08
π_3, 1.81
π_4, 1.63
π_5, 1.45
π_3^*, 0.19
π_4^*, 0.26
π_5^*, 0.70
b. FlH$^{\cdot}$
π_n, 1.98
$\pi_{C2=O}$, 1.95
$\pi_{C4=O}$, 1.94
π_1, 1.95
π_2, 1.80
$\pi^*_{C2=O}$, 0.04
$\pi^*_{C4=O}$, 0.07
π_1^*, 0.04
π_3, 1.75
π_4, 1.66
π_5, 1.26
π_2^*, 0.10
π_3^*, 0.16
π_4^*, 0.29

c. Fl$^{\bullet-}$

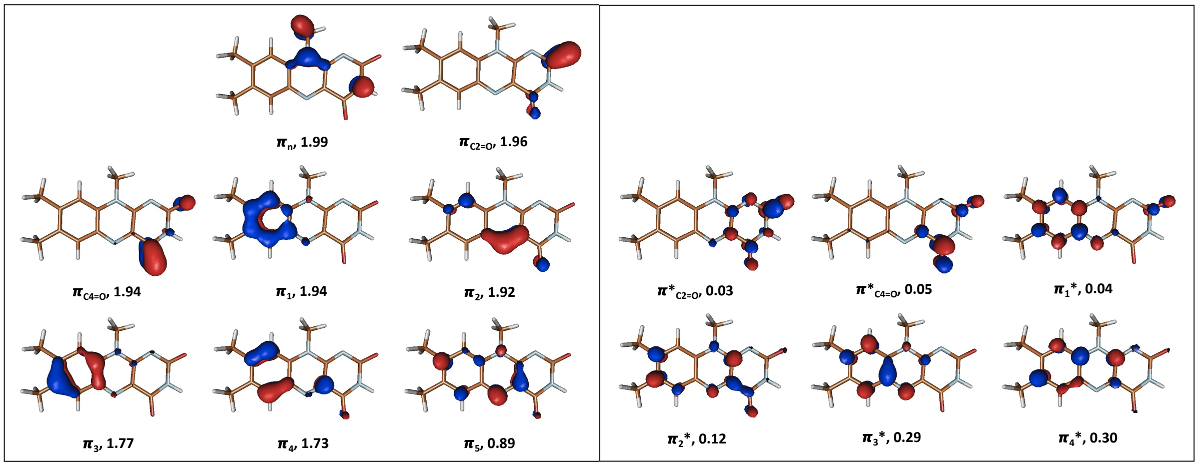

d. FlH$_2$

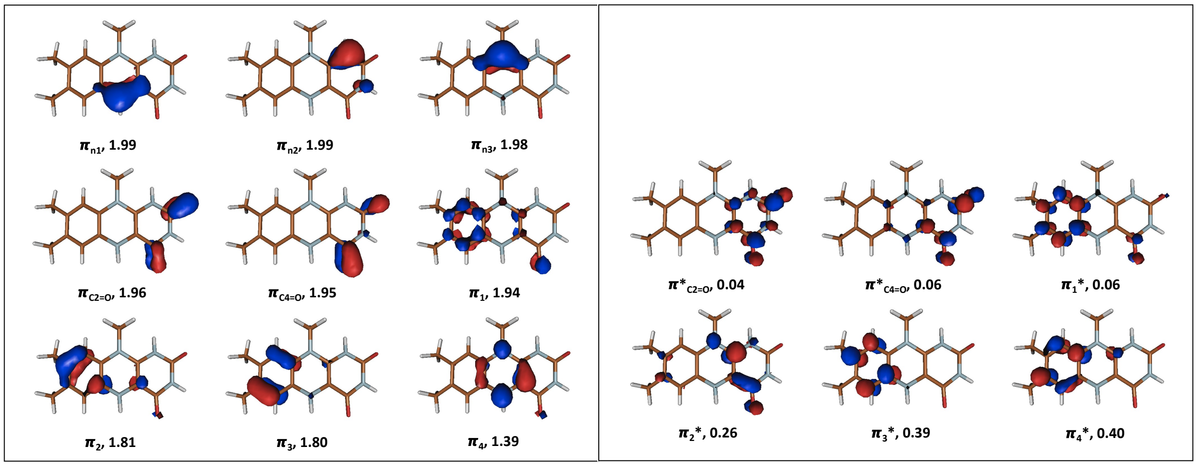

e. FlH$^-$

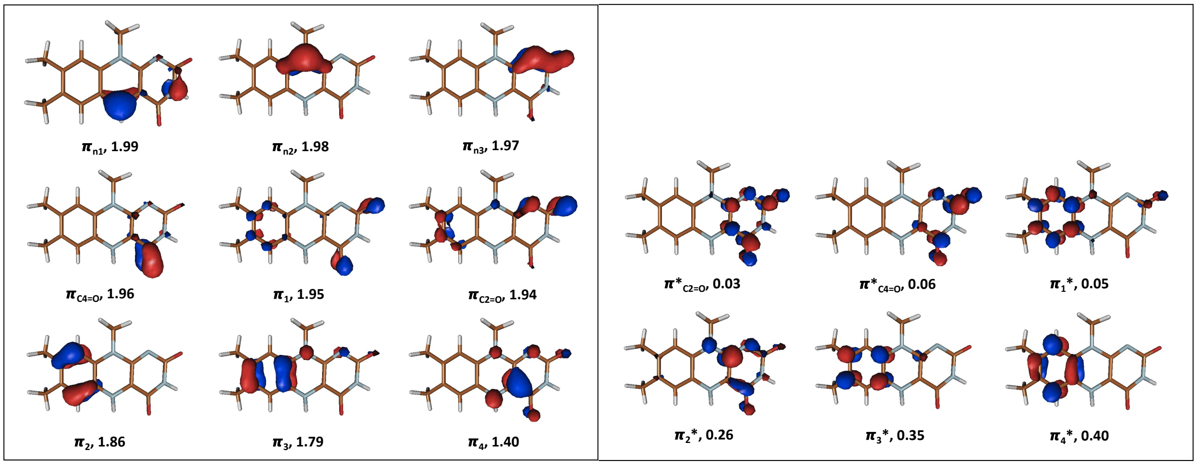

Figure S1. Occupied (left) and vacant (right) orbitals used as a starting point for the active space of flavin in each of five redox and protonation states. **a.** oxidized quinone (Fl), **b.** neutral semiquinone radical (FlH$^{\bullet}$), **c.** anionic semiquinone radical (Fl$^{\bullet-}$), **d.** neutral hydroquinone (FlH$_2$), and **e.** anionic hydroquinone (FlH$^-$).

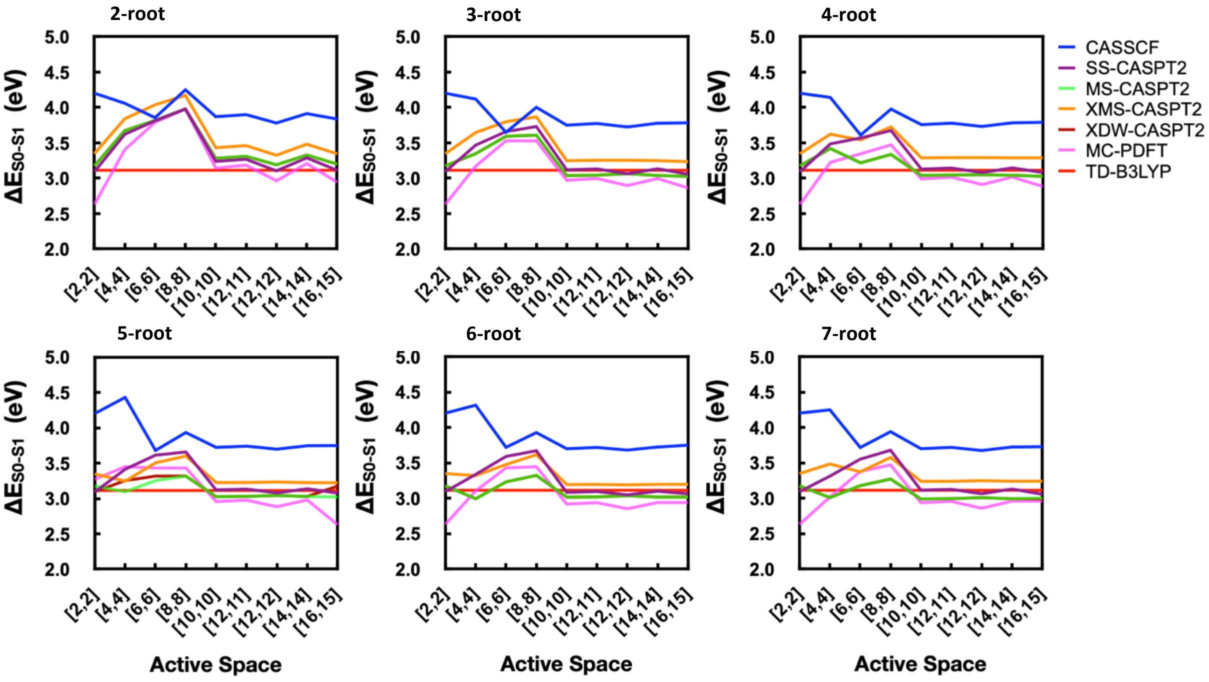

Figure S2. MR-PT2 and CASSCF S_1 excitation energies as a function of active space [number of electrons, number of orbitals] with 2-root averaging, 3-root state averaging, 4-root state averaging, 5-root state averaging, 6-root state averaging, and 7-root state averaging for LF in the oxidized form. The TD-B3LYP excitation energy is shown as a red line for reference.
